# The complete mitochondrial genome of *Bothrogonia qiongana* (Hemiptera: Cicadellidae) with phylogenetic analyses

**DOI:** 10.1080/23802359.2020.1788437

**Published:** 2020-07-14

**Authors:** Xiao-Li Xu, Bin Yan, Xiao-Fei Yu, Mao-Fa Yang

**Affiliations:** a College of Agriculture, Guizhou University, Guiyang, China; b Guizhou Provincial Key Laboratory for Agricultural Pest Management of the Mountainous Region, Institute of Entomology, Guizhou University, Guiyang, China; c College of Tobacco Science, Guizhou University, Guiyang, China; d Guizhou Key Laboratory of Tobacco Quality Research, Guiyang, Guizhou, China

**Keywords:** Mitochondrial genome, *Bothrogonia qiongana*, phylogenetic analyses, Cicadellidae

## Abstract

The species of *Bothrogonia qiongana* was reported by Yang and Li in 1980. The mitogenome is 15,788 bp in length, including 37 genes and a control region, with an overall A + T content of 76.9%. Most of the PCGs start with ATN (ATA, ATC, ATT, ATG), but ND5 starts with TTG. All PCGs used TAA or TAG as stop codon except COX2 which is using incomplete single T––. The phylogenetic tree was reconstructed based on *B. qiongana* and 24 reference species, which is consistent with traditional taxonomy.

The genus *Bothrogonia* China was established and reviewed in the twentieth century (Melichar 1926; China 1938; Yang and Li 1980). Totally 47 species were recorded in the world (Yang et al. 2017). The mitochondrial genome of *B. ferruginea* was reported firstly (Yu et al. 2019). In this study, the complete mitochondrial genome of *B. qiongana* was sequenced and annotated, and the phylogeny analysis was revealed based on 25 species in Cicadellidae.

Total DNA was extracted from a male adult which was collected from Bawangling National Nature Reserve in Hainan Province of China (19° 4′52.6224″N, 109°11′31.8984″E) on 9 April 2017. The male genitalia are deposited in the Institute of Entomology, Guizhou University, Guiyang, China (GUGC), and the deposited number is GUGC-IDT-00200. Then, the mitogenome of *B. qiongana* was sequenced by Illumina NovaSeq6000 platform (Berry Genomics, Beijing, China). The raw data were assembled and annotated by NOVOPlasty, MitoZ and Generous Prime. All tRNA genes were identified by ARWEN online service. The annotated sequences was submitted to GenBank with accession number MT500855. The phylogeny tree was reconstructed based on amino acid sequences of 13 PCGs among *B. Qiongana* and 24 reference species, using the partition model determined by maximum likelihood method in IQ-TREE software (Figure 1). The tree was visualized in FigTree v.1.4.2 (Rambaut 2016) and edited using Adobe Illustrator CC 2018.

**Figure 1.**
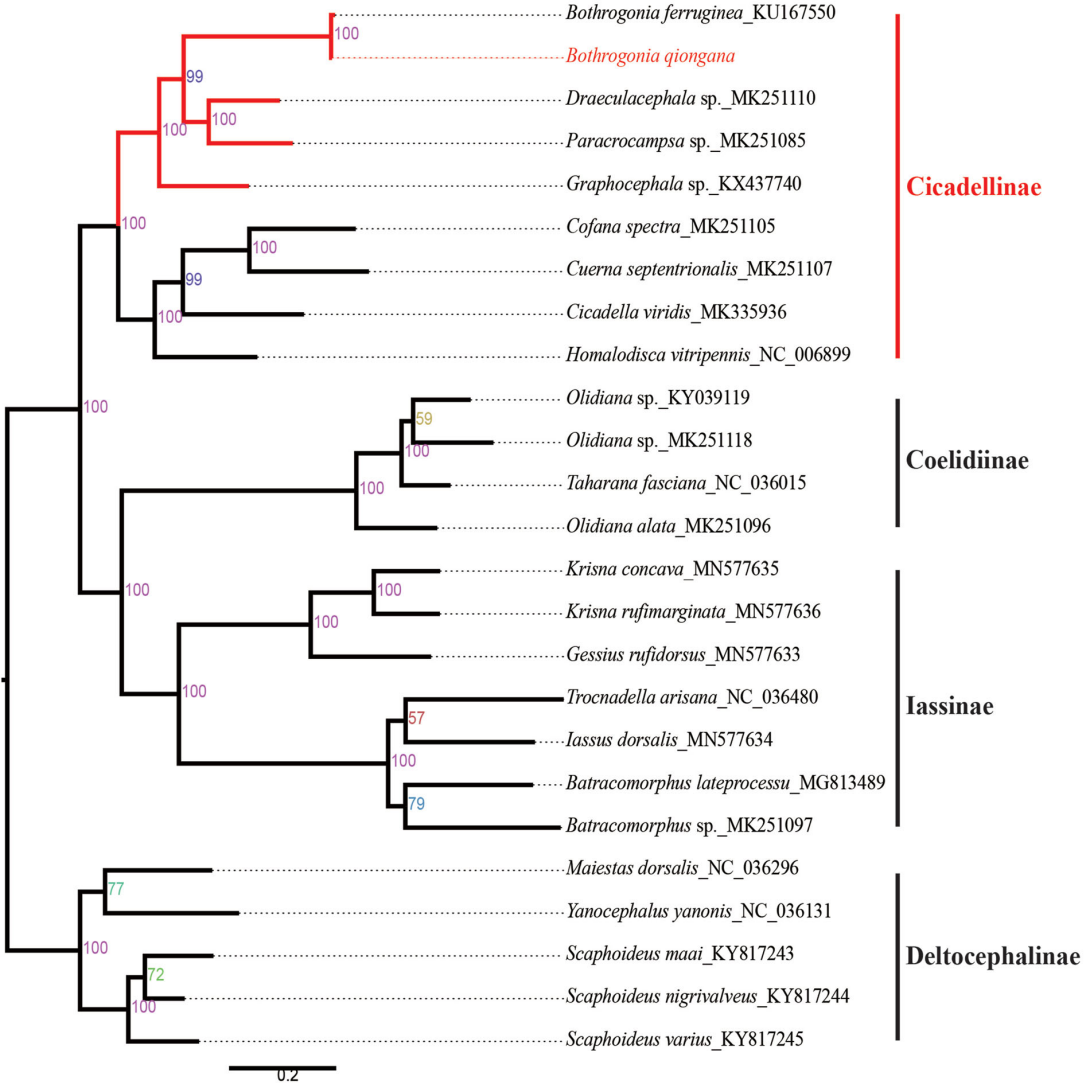
Phylogenetic analyses of *Bothrogonia qiongana* based on the amino acid sequences of the 13 PCGs. Numbers at nodes are bootstrap values. The GenBank accession number for each species is indicated after the scientific name.

The species of *B. qiongana* is 15,788 bp long, containing 13 protein-coding genes (PCGs), 22 transfer RNA genes (tRNA), 2 ribosomal RNA genes (rRNA), and a contol region (C-region). The base composition values for *B. qiongana* mitogenome were 44.8, 13.1, 10.0, and 32.1% for A, C, G, and T, respectively, with an overall A + T content of 76.9%. Most of the PCGs start with ATN (ATA, ATC, ATT, ATG), but ND5 starts with TTG. All PCGs used TAA or TAG as stop codon except COX2 which is using incomplete single T. All the tRNAs are between 60 bp (tRNA-R) and 73 bp (tRNA-M) in size. The length of 16S rRNA and 12S rRNA gene are 1155 bp and 774 bp. The species of *B. qiongana* and *B. ferruginea* is clustered into one clade, which is consistent with traditional taxonomy. This study benefit research on population genetics and evolution of Cicadellidae.

## Data Availability

The data that support the findings of this study are openly available in GenBank of NCBI at https://www.ncbi.nlm.nih.gov reference number MT500855.
